# Preoperative intra-articular steroid injections within 3 months increase the risk of periprosthetic joint infection in total knee arthroplasty: a systematic review and meta-analysis

**DOI:** 10.1186/s13018-023-03637-4

**Published:** 2023-02-28

**Authors:** Young Mo Kim, Yong Bum Joo, Ju-Ho Song

**Affiliations:** 1 Department of Orthopedic Surgery, Chungnam National University Hospital, Chungnam National University College of Medicine, Daejeon, Republic of Korea; 2 Department of Orthopedic Surgery, Chungnam National University Sejong Hospital, Chungnam National University College of Medicine, Sejong, 30099 Republic of Korea

**Keywords:** Total knee arthroplasty, Injection, Steroid, Periprosthetic joint infection

## Abstract

**Objective:**

Albeit with no disease-modifying effects, intra-articular steroid injections (IASIs) are still widely used to relieve symptoms of knee osteoarthritis. Previous literature has reported conflicting results regarding the safety of IASI in terms of periprosthetic joint infection (PJI) in total knee arthroplasty (TKA). This study tried to determine whether preoperative IASIs increased the risk of PJI, with different time intervals between the injections and surgery.

**Methods:**

A computerized search of MEDLINE, EMBASE, and Cochrane Library was conducted for studies published before October 2022, which investigated the PJI rates of patients who received IASIs before TKA and patients who did not. The primary outcome was the association between preoperative IASI and PJI in TKA. The time point from which IASIs could be applied without risking PJI was also assessed.

**Results:**

Fourteen studies, with 113,032 patients in the IASI group and 256,987 patients in the control group, were included. The pooled odds ratio of PJI was 1.13 (95% confidence interval [CI] 1.00–1.27, p = 0.05), indicating no increased risk of PJI. With the time interval < 6 months, the pooled odds ratio was 1.19 (95% CI 0.99–1.43, p = 0.06). However, with the time interval < 3 months, the pooled odds ratio was 1.26 (95% CI 1.06–1.50, p < 0.01).

**Conclusion:**

IASI is not a safe procedure for patients who are expected to undergo TKA. The time interval between the injections and surgery was an important factor in assessing the safety of IASI. Preoperative injections that were applied within 3 months increased the risk of PJI in TKA.

**Supplementary Information:**

The online version contains supplementary material available at 10.1186/s13018-023-03637-4.

## Introduction

Osteoarthritis (OA) of the knee is one of the most common causes of musculoskeletal disability worldwide. The life time risk of symptomatic knee OA has been reported to be about 40% [1–3]. However, there are no available disease-modifying treatments [4] and instead, the current guidelines relied on traditional analgesics and non-pharmacologic interventions for pain relief [5, 6]. Nearly 30% of patients received intra-articular steroid injections (IASIs) before undergoing total knee arthroplasty (TKA) [7].

Several medications are used in intra-articular injection, including corticosteroid, hyaluronic acid, platelet-rich plasma, and stem cells [8, 9]. Although widely applied, the medications have showed limited evidence for the efficacy in treating OA of the knee [10, 11]. The risk benefit of each medication should be evaluated carefully, considering the inherent risk of intra-articular injection such as periprosthetic joint infection (PJI).

PJI is a devastating complication in TKA, occurring at a rate of 0.5%–1.8% [12, 13]. Unfortunately, a controversy remains unresolved regarding whether preoperative IASIs increase the risk of PJI [4, 14–16] or not [17–19]. The low incidence of PJI has been an obstacle in designing a robust study. Some studies included patients who underwent knee joint arthroplasty and patients who underwent hip joint arthroplasty together in assessing the risk of PJI [20–22]. Some authors did not specify medications injected into the joint (corticosteroid, hyaluronic acid, or others) [23, 24]. The previous studies have investigated the risk of PJI in the setting of various time intervals (from 4 weeks to 1 year) between preoperative injections and arthroplasty [25–27]. These inconsistencies compromised the reliability of the conclusions.

The American Academy of Orthopedic Surgeons (AAOS) has recently downgraded its recommendation on IASI [5]. If the aforementioned controversy is resolved, the role of IASI can be established in a spectrum of treatments for knee osteoarthritis. A meta-analysis is expected to compensate the inconsistencies of each primary study. This study tried to determine whether preoperative IASIs increased the risk of PJI in TKA, with different time intervals between the injections and surgery.

## Methods

The present meta-analysis was written according to the Preferred Reporting Items for Systemic Reviews and Meta-Analyses guidelines [28]. Ethical approval and acquisition of informed consent from participants were not required because all data were based on already published studies and were anonymously analyzed without any potential harm to the participants.

### Literature search

A computerized search of MEDLINE, EMBASE, and Cochrane Library was conducted for studies published before October 2022, which investigated the PJI rates of patients who received IASIs before TKA and patients who did not. The search query included synonyms for total knee arthroplasty, steroid, injection, infection, and complication as follows: ([arthroplasty, replacement, knee] OR [total knee arthroplasty]) AND ([steroids] OR [adrenal cortex hormones] OR [corticosteroid]) AND [injections] AND ([safety] OR [infection] OR [complication]). The search was confined to studies on “humans” in the “English” language. Bibliographies of the studies were checked to identify additional relevant studies.

### Inclusion and exclusion criteria

The included studies fulfilled the following criteria: (1) patients diagnosed with PJI after TKA; (2) patients who had a history of preoperative IASI; (3) follow-up duration more than six months to determine PJI and sufficient data to tabulate 2 × 2 contingency tables for odds ratios; and (4) publication type of original articles. We excluded studies with the following criteria: (1) patients who had arthroplasty other than primary TKA, such as revision TKA, unicompartmental knee arthroplasty, and total hip arthroplasty; (2) intra-articular injection applied perioperatively for pain control purpose; (3) outcomes that did not include PJI rates; and (4) review articles, editorials, letters, and single case studies. The above process was independently performed by two reviewers with consultation from a third reviewer for reaching a consensus when any disagreements were present.

### Data extraction and quality assessment

Two reviewers independently extracted data from each study using a standardized data extraction form: patient characteristics such as the size of study population, the number of patients with PJI, and the time points of preoperative IASIs; and study characteristics including authors, institutions, publication year, study design, and follow-up duration.

The risk of bias was assessed using the Cochrane-recommended Risk of Bias in Non-randomized Studies of Intervention (ROBINS-I) tool because all included studies were non-randomized. The ROBINS-I tool provides signaling questions for reviewers to determine low, moderate, serious, or critical risk of bias among evaluated studies [29].

### Data synthesis and analyses

The primary outcome of this study was the association between IASI and PJI. The time point from which IASIs could be applied without risking PJI was also assessed. Two by two tables were made for the odds ratio of PJI in association with IASI. As most primary studies reported time points of IASIs with 3-month intervals, the odds ratios were assessed with the time interval < 3 months and the time interval < 6 months. Although a few studies investigated both superficial infection and deep infection, only the latter was counted in the present study because diagnostic standard for superficial infection was unclear; many factors could affect the incidence of superficial infection; and it was intra-articular infection that led to devastating outcomes.

Summary estimates of odds ratios were calculated with a random-effects model to avoid overestimation of the study results. Heterogeneity was evaluated with forest plots and was quantified by Higgins I^2^ test, in which 25%, 50%, and 75% were considered as low, moderate, and high heterogeneities, respectively [30]. All statistical analyses were performed using the Review Manager (RevMan) program Version 5.4.1 (The Nordic Cochrane Center, The Cochrane Collaboration, 2014; Copenhagen Denmark).

## Results

The electronic search query applied in MEDLINE, EMBASE, and Cochrane Library is summarized in Additional file 1.

**Fig. 1:**
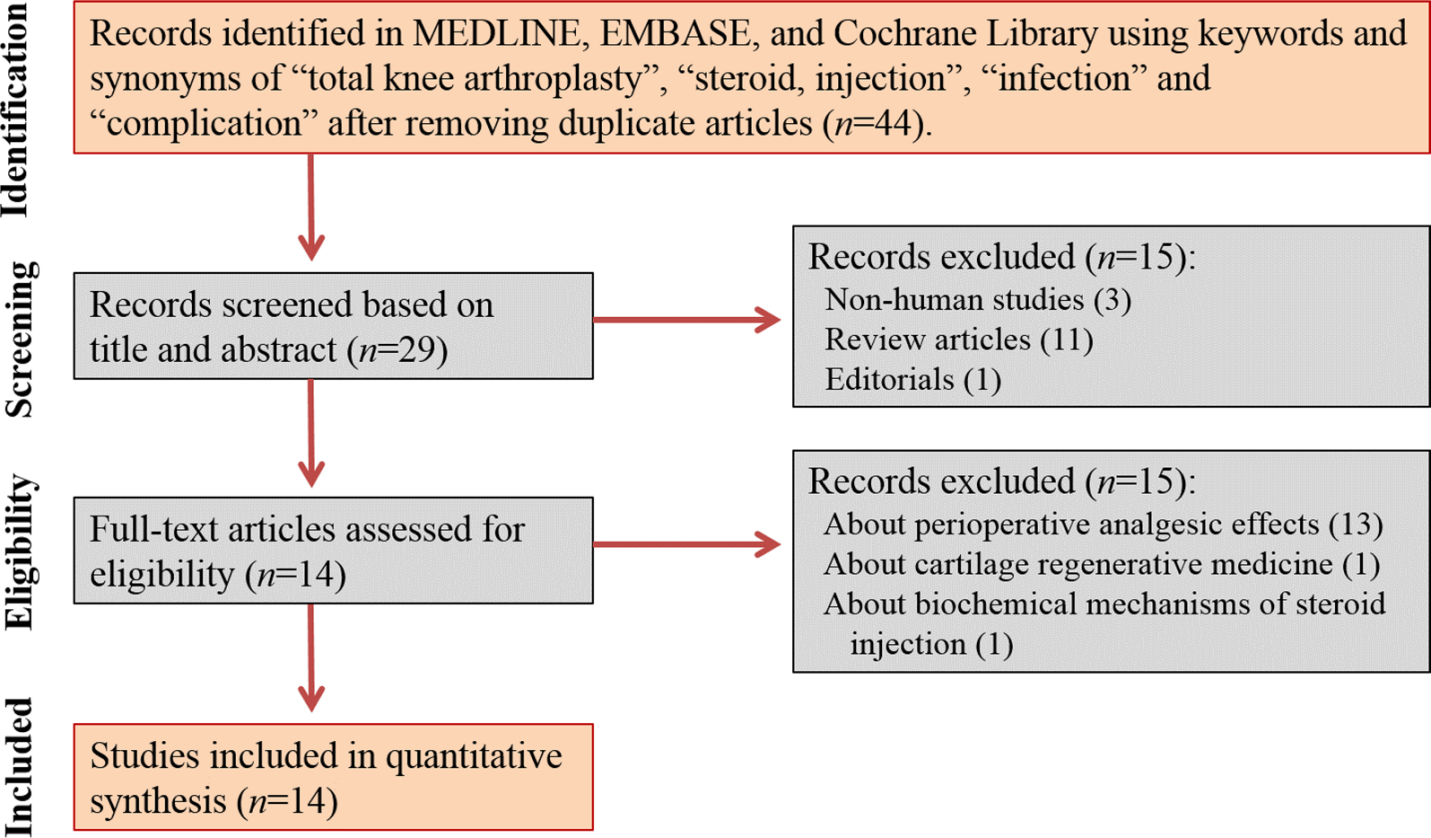
Preferred reporting items for systematic reviews and meta-analyses (PRISMA) flow chart

Of those 44 articles, we removed three non-human studies and additionally ruled out twelve studies after screening titles and abstracts, which left 29 original articles. Based on full text reviews, fifteen studies were not found to be in the field of interest: thirteen studies about perioperative injection for analgesic effects; one study about the injection of cartilage regenerative medicine; and one study about cytokine-related mechanisms following steroid injection. Accordingly, fourteen studies were included in the final analysis (Fig. 1).

### Study characteristics

A total of 370,019 patients were analyzed. Of these, there were 113,032 patients in the IASI group and 256,987 patients in the control group. Nine studies investigated the time intervals between IASIs and TKA [4, 15–17, 19, 25, 27, 31, 32]. In making the diagnosis of PJI, seven studies were based on microbiologic culture results and the other seven studies relied on the relevant disease codes, such as the Current Procedural Terminology code and the International Classification of Diseases 9th Revision code regarding postoperative infections and operative procedures to address them [4]. The characteristics of the included studies are summarized in Table 1.

**Table 1:** Characteristics of the included studies

Authors	Year	Mean age	Sex (M/F)	Sample size	PJI rate (n/N)		Criteria for PJI
					IASI group	Control group	
Amin et al. [19]	2015	63.82	N/A	1,628	4/783	12/845	MSIS criteria
Bedard et al. [4]	2017	N/A	30,162/53,522	83,684	403/29,603	563/54,081	Disease codes
Bhattacharjee et al. [25]	2021	N/A	28,081/48,789	76,970	63/8,226	696/68,744	Disease codes
Cancienne et al. [15]	2015	N/A	11,340/24,550	35,890	949/22,240	500/13,650	Disease codes
Desai et al. [33]	2009	68	100/150	270	0/90	0/180	Microbiologic culture
Grondin et al. [34]	2021	71.8	84/220	180	4/83	2/97	Microbiologic culture
Khan et al. [35]	2022	69.5	645/675	1,169	7/521	15/648	Disease codes
Khanuja et al. [17]	2016	66	164/440	604	10/302	12/302	CDC criteria
Kurtz et al. [31]	2021	N/A	32,678/57,159	89,837	273/33,331	424/56,506	Disease codes
Papavasiliou et al. [14]	2006	N/A	N/A	144	3/54	0/90	Microbiologic culture
Rhode et al. [32]	2021	61.33	104/313	367	24/189	15/178	Microbiologic culture
Richardson et al. [16]	2019	N/A	17,244/28,487	58,337	532/16,656	1,117/41,681	Disease codes
Tang et al. [36]	2021	63.9	751/1,018	1,429	0/51	7/1,378	Medical records
Turcotte et al. [27]	2020	66.5	N/A	19,510	28/903	416/18,607	Disease codes

PJI, periprosthetic joint infection; IASI, intra-articular steroid injection; N/A, not available; MSIS, musculoskeletal infection society; CDC, centers for disease control and prevention

### Risk of bias assessment

**Fig. 2:**
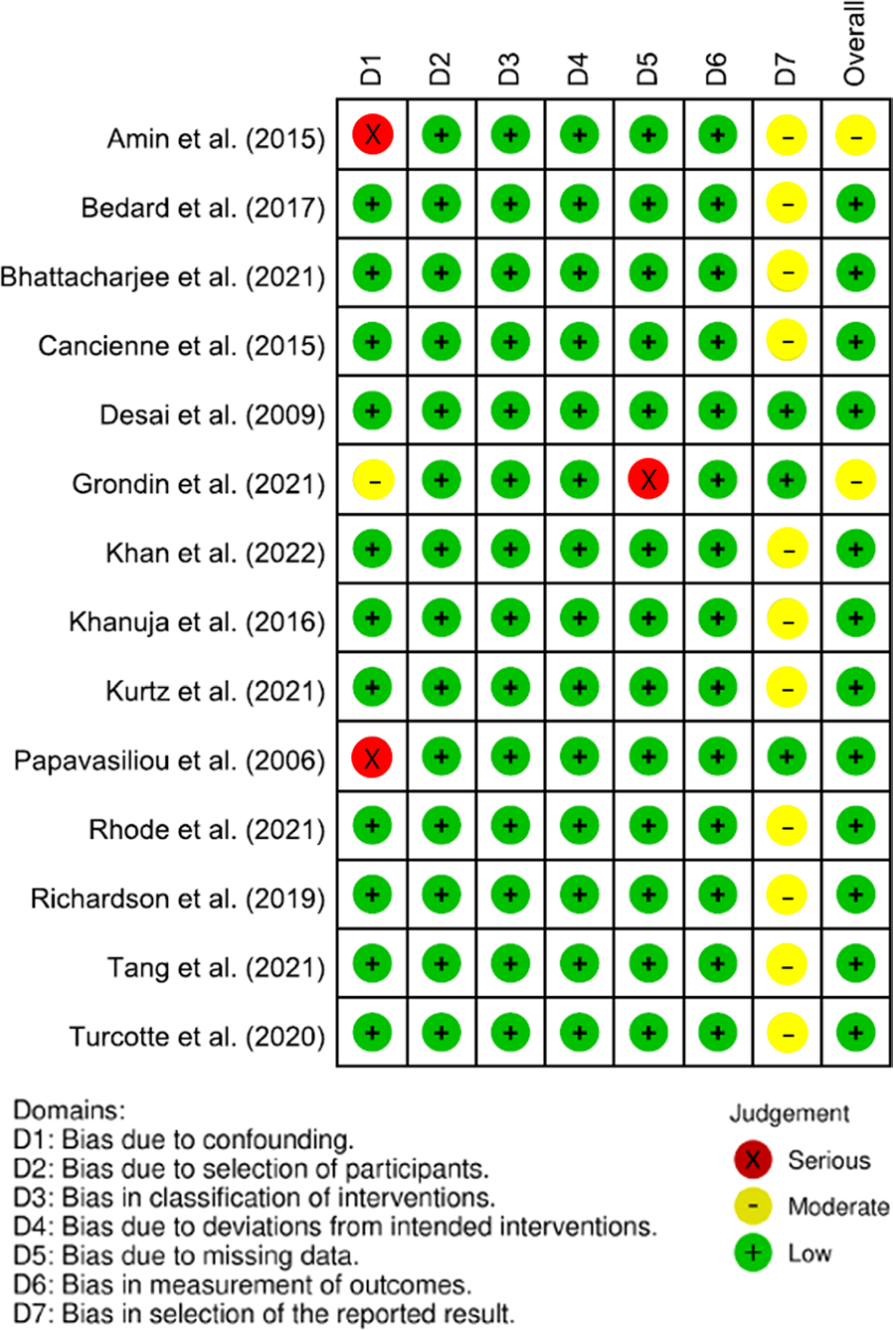
Traffic light plot regarding risk of bias scores for the included studies

The overall risk of bias was assessed to be low in twelve studies based on ROBINS-I assessment [4, 14–17, 25, 27, 31–33, 35, 36]. Moderate risk of bias was assigned to two studies [19, 34]. However, no studies were determined to be at serious risk of bias (Fig. 2).

### Risk of PJI with different time intervals between IASIs and TKA

**Fig. 3:**
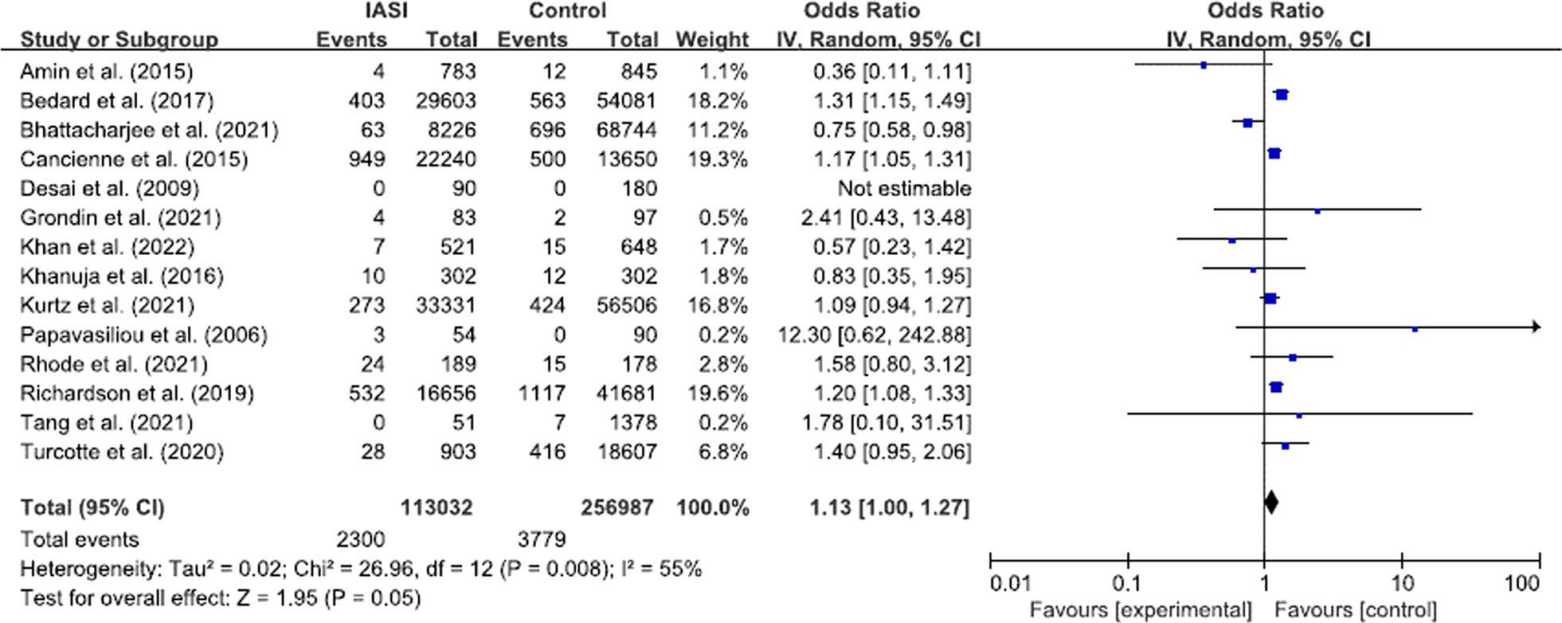
Forest plot regarding the overall effect of IASI on the risk of PJI. IASI, intra-articular steroid injection; PJI, periprosthetic joint infection

Overall, 2,300 (2.0%) PJIs were noted in the IASI group and 3,779 (1.5%) PJIs were reported in the control group. The pooled odds ratio of PJI was 1.13 (95% confidence interval [CI] 1.00–1.27, p = 0.05, I^2^ = 55%; Fig. 3), which indicated no increased risk of PJI.

**Fig. 4:**
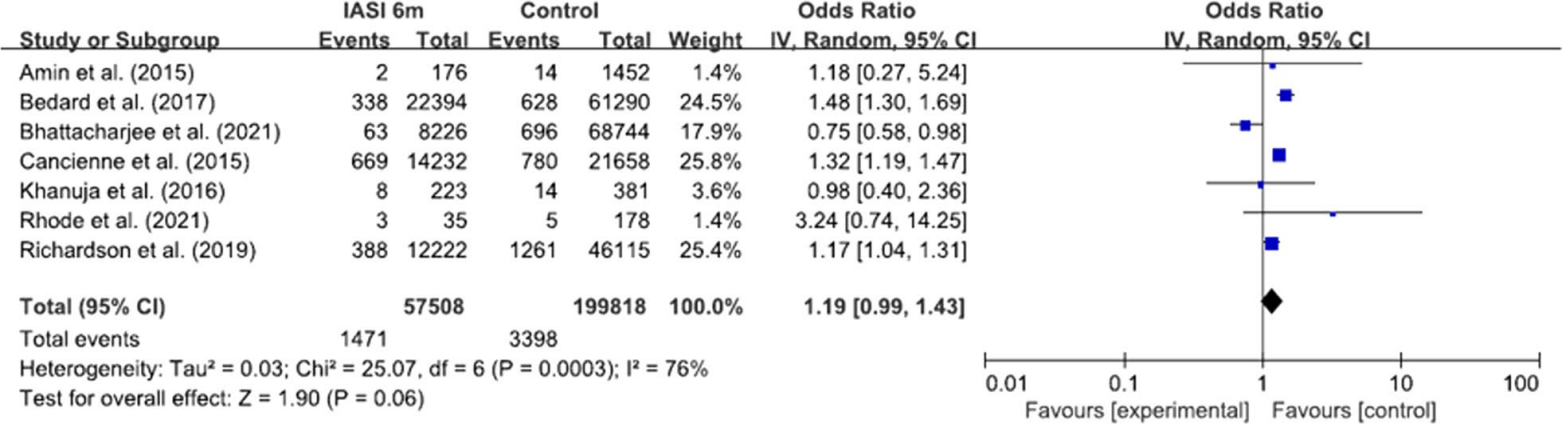
Forest plot regarding the effect of IASI within 6 months on the risk of PJI. IASI, intra-articular steroid injection; PJI, periprosthetic joint infection

**Fig. 5:**
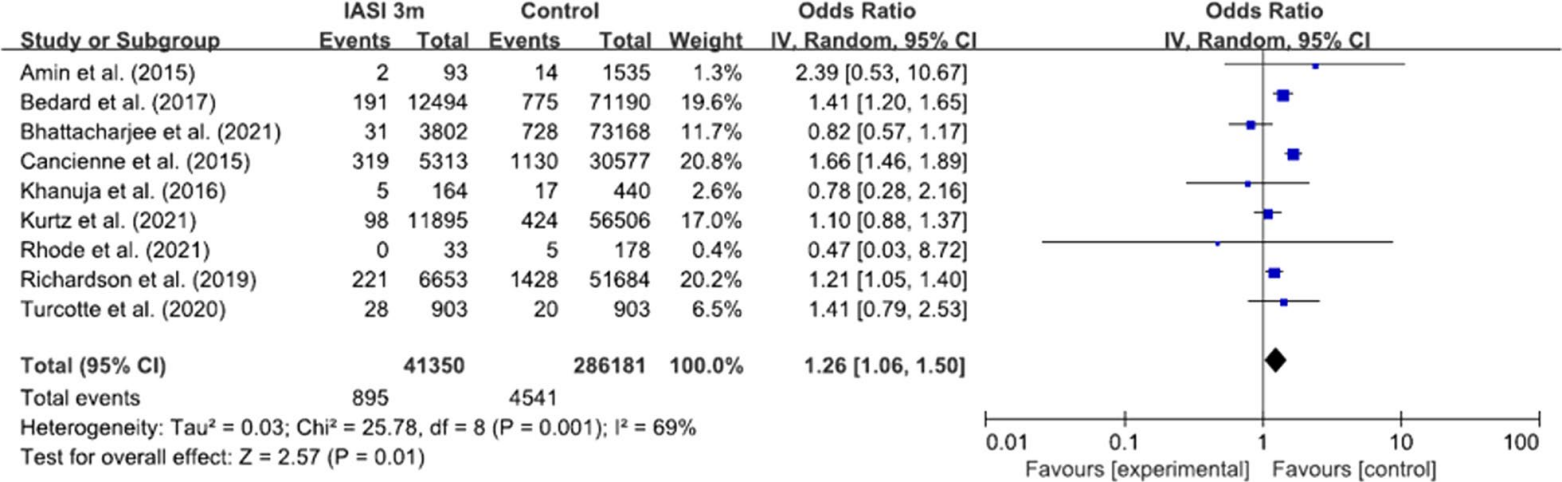
Forest plot regarding the effect of IASI within 3 months on the risk of PJI. IASI, intra-articular steroid injection; PJI, periprosthetic joint infection

Nine studies reported regarding the time intervals between preoperative injections and surgery [4, 15–17, 19, 25, 27, 31, 32]. With the time interval < 6 months, the pooled odds ratio was 1.19 (95% CI 0.99–1.43, p = 0.06, I^2^ = 76%; Fig. 4). However, with the time interval < 3 months, the pooled odds ratio was 1.26 (95% CI 1.06–1.50, p < 0.01, I^2^ = 69%; Fig. 5).

## Discussion

The most important finding of this study was that preoperative IASIs had a significant association with PJI when the time interval between the injections and surgery was considered. IASIs that were applied within 3 months increased the risk of PJI in TKA. Because the time interval of 3–6 months has not been investigated enough in the literature, it is safe to keep 6-month interval between IASIs and TKA.

Previous studies have reported inconsistent results regarding the association between preoperative IASI and TKA. Primary studies that could not find the association had a small sample size [14, 17, 19, 32–34, 36]. Due to the low incidence of PJI, those studies were likely to be underpowered and bore the risk of type 2 error. Studies that were based on large database [4, 15, 25, 31] or collected an enough study population [16] proved the increased risk of PJI when IASIs were applied preoperatively. A large database study by Cancienne et al., however, had a limitation in that the database was confined to a Medicare-only population [15], making it difficult to apply their results to the younger population [4]. Recent meta-analyses did not distinguish injected medications in assessing the risk of PJI [23, 24].

Specification of injected medications is crucial in establishing practical guidelines of osteoarthritis treatments. Current guidelines present recommendations separately for each medication. The intra-articular corticosteroid recommendation has been downgraded because of potential risk in accelerating osteoarthritis [5]. Despite its short-term effect for symptomatic knee osteoarthritis, intra-articular corticosteroid injection was associated with subchondral insufficiency fracture, osteonecrosis, and rapid bone loss [37]. Knee osteoarthritis is a progressive disease, and unfortunately no disease-modifying treatments are available to date [38, 39]. Because non-surgical managements and TKA should be considered together in treating knee osteoarthritis, the safety time point from which IASI could be performed without risking PJI needs to be determined.

Nine studies have investigated the safety time point, reporting different results [4, 15–17, 19, 25, 27, 31, 32]. A recent national database study by Bhattacharjee et al. stratified the time interval between IASIs and TKA into biweekly cohorts. It concluded that TKA performed within 4 weeks of IASI was associated with a higher risk of PJI [25]. Bedard et al. classified the injection cohort by monthly intervals and found that the odds of PJI remained higher for the injection cohort out to a duration 6 months between injections and TKA [4]. Other two studies also reported that the safety time point was 6 months [15, 16]. The present meta-analysis investigated the time point with 3-month interval. The pooled analysis did not show the increased risk of PJI within 6 months; however, the odds of PJI were significantly increased within 3 months of IASI.

Several limitations should be noted. First, the primary studies included in this meta-analysis were retrospective studies. Because of its low incidence, the risk of PJI has not been investigated in randomized controlled trials. Second, there was moderate heterogeneity that could not be fully addressed although a random-effects model was adopted. Six studies utilized large insurance-related database. Accordingly, the diagnosis of PJI was made using the relevant disease codes, whereas the diagnosis was based on microbiologic culture results in the other studies. Third, other potential risk factors of PJI could not be investigated. The number of intra-injections [26] and patient’s demographics might also be important factors. Further research is required to fully understand the association between IASI and PJI. Fourth, the mechanism of how IASIs lead to PJI could not be elucidated. It might be the result of mechanical inoculation or the chemical influence of intra-articular steroid. However, this study could not investigate the mechanism, which is an inherent limitation of meta-analyses.

## Conclusions

IASI is not a safe procedure for patients who are expected to undergo TKA. The time interval between the injections and surgery was an important factor in assessing the safety of IASI. Preoperative injections that were applied within 3 months increased the risk of PJI in TKA.

## Supplementary Information

**Additional file 1.** The electronic search query applied in MEDLINE, EMBASE, and Cochrane Library.

## Data Availability

The datasets generated and analyzed during the current study are available from the corresponding author on reasonable request.
