# Supplementary material for: Preoperative intra-articular steroid injections within 3 months increase the risk of periprosthetic joint infection in total knee arthroplasty: a systematic review and meta-analysis
Source: J Orthop Surg Res. 2023 Feb 28;18:148. doi: 10.1186/s13018-023-03637-4 (PMC9972619; doi:10.1186/s13018-023-03637-4)
Supplement: Supplementary file 1 — Additional file 1. The electronic search query applied in MEDLINE, EMBASE, and Cochrane Library. [file 13018_2023_3637_MOESM1_ESM.docx]

Supplementary file 1. The electronic search query in the meta-analysis

#1 "arthroplasty, replacement, knee"[MeSH Terms or Emtree]; 18989

#2 "total knee arthroplasty"[TIAB]; 14785

#3 #1 OR #2; 23771

#4 steroids[MeSH Terms or Emtree]; 808488

#5 steroids[TIAB]; 85034

#6 #4 OR #5; 848712

#7 "injections"[MeSH Terms or Emtree]; 269754

#8 "injections"[TIAB]; 135242

#9 #7 OR #8; 367144

#10 #3 AND #6 AND #9; 37

#11 "adrenal cortex hormones"[MeSH Terms or Emtree]; 268629

#12 corticosteroid[TIAB]; 92624

#13 #6 OR #11 OR #12; 953916

#14 #3 AND #9 AND #13; 69

#15 "safety"[MeSH Terms or Emtree] OR "patient safety"[MeSH Terms or Emtree]; 70480

#16 “infection”[MeSH Terms or Emtree]; 730834

#17 complication[TIAB]; 854096

#18 safety[TIAB]; 413373

#19 infection[TIAB]; 1238516

#20 #15 OR #16 OR #17 OR #18 OR #19; 2766363

#21 #14 AND #20; 44
